# Integration of RNA molecules data with prior-knowledge driven Joint Deep Semi-Negative Matrix Factorization for heart failure study

**DOI:** 10.3389/fgene.2022.967363

**Published:** 2022-10-10

**Authors:** Zhihui Ma, Bin Chen, Yongjun Zhang, Jinmei Zeng, Jianping Tao, Yu Hu

**Affiliations:** Department of Cardiology, Shanghai Sixth People's Hospital Affiliated to Shanghai Jiao Tong University School of Medicine, Shanghai, China

**Keywords:** heart failure, biomarker, joint nonnegative matrix factorization, diagnostic model, machine learning

## Abstract

Heart failure (HF) is the main manifestation of cardiovascular disease. Recent studies have shown that various RNA molecules and their complex connections play an essential role in HF’s pathogenesis and pathological progression. This paper aims to mine key RNA molecules associated with HF. We proposed a Prior-knowledge Driven Joint Deep Semi-Negative Matrix Factorization (PD-JDSNMF) model that uses a hierarchical nonlinear feature extraction method that integrates three types of data: mRNA, lncRNA, and miRNA. The PPI information is added to the model as prior knowledge, and the Laplacian constraint is used to help the model resist the noise in the genetic data. We used the PD-JDSNMF algorithm to identify significant co-expression modules. The elements in the module are then subjected to bioinformatics analysis and algorithm performance analysis. The results show that the PD-JDSNMF algorithm can robustly select biomarkers associated with HF. Finally, we built a heart failure diagnostic model based on multiple classifiers and using the Top 13 genes in the significant module, the AUC of the internal test set was up to 0.8714, and the AUC of the external validation set was up to 0.8329, which further confirmed the effectiveness of the PD-JDSNMF algorithm.

## Introduction

Heart failure (HF) is a common type of cardiovascular disease, and its morbidity and mortality are increasing yearly (Castiglione et al., 2022). The pathogenesis of HF involves multiple risk factors, especially diabetes, acute myocardial infarction, hypertension, and coronary heart disease (Fan and Hu, 2022). Despite significant improvements in disease diagnosis and treatment, the prognosis of patients with HF remains poor (Gomes et al., 2020). Various methods have been used to diagnose HF, such as echocardiography, clinical signs, and NT-proBNP/BNP. However, these methods have certain limitations. For example, echocardiography relies on experts’ operation ability and rich experience. NT-proBNP/BNP is an invasive diagnosis and may harm HF patients. Therefore, genes or other genetic material have emerged as an alternative non-invasive method for disease diagnosis (Chair et al., 2021). In addition, genotypic biomarkers also provide potential targets for drug development. The search for new biomarkers is of great significance for improving the diagnosis and treatment of HF.

With the development of RNA sequencing data, researchers can effectively identify and mine disease-related RNA molecular biomarkers. In addition to messenger RNA (mRNA), long non-coding RNA (lncRNA) and microRNA (miRNA) have also been shown to be extensively involved in the pathological progression of HF (Fan et al., 2018). MiRNAs can alter cardiac differentiation, proliferation, maturation, and pathological remodeling responses (Yan et al., 2017). Sala et al., 2016 reviewed several miRNAs that play an essential role in HF, such as miR-18a-5p, miR-652–3p, and miR-126 (Wang and Cai, 2017). LncRNAs are essential regulators during cardiovascular development (Fan et al., 2018). Researchers found that lncRNA-Cancer Susceptibility Candidate 7 (CASC7) is involved in the progression of HF by regulating the expression of miR-30c, which is also a promising diagnostic-related gene for HF (Xu et al., 2020). In addition, the three RNA molecules are complexly interconnected (Song et al., 2016). Therefore, identifying HF-related RNA molecules may provide new insights into the pathogenesis and progression of HF.

Due to their complementary information, multi-omics data can capture and mine disease-related and biologically meaningful biomarkers. Exploring multi-omics integration algorithms is a hot topic in bioinformatics research. Joint non-negative matrix factorization (JNMF) has attracted the attention of researchers due to its low time complexity and strong clustering performance. Zhang et al. first proposed this algorithm, applied it to cancer genomics (Zhang et al., 2012), and identified multiple biologically meaningful co-expression modules. Deng et al. used this algorithm to construct a ceRNA network closely related to lung cancer (Deng et al., 2018). Further, they added orthogonal constraints to the algorithm and proposed a Multi-Constrained Joint Non-negative Matrix Factorization (MCJNMF) algorithm. The algorithm integrates PET image data and DNA methylation data of patients with soft tissue sarcoma and mines biomarkers and significant imaging features related to soft tissue sarcoma lung metastasis (Deng et al., 2019). Recently, to integrate pathological images of soft tissue sarcomas with two genetic data (DNA methylation and copy number variation), they proposed a Multi-Dimensional Joint Non-negative Matrix Factorization (MDJNMF) algorithm that integrates multiple biological empirical knowledge, the potential association pattern with the three kinds of data was found through multi-level analysis. The comprehensive prediction index AUC of the identified relevant biomarkers reached 0.8 (Deng et al., 2021). The above matrix factorization correlation algorithms are based on linear assumptions and cannot consider the complex relationship between multi-omics data from a nonlinear perspective. To this end, Sehwan Moon et al. proposed a Joint Deep Semi-Negative Matrix Factorization (JDSNMF) algorithm, which applies a deep neural network (DNN) to the JNMF algorithm to identify disease-related significant nonlinear features. However, the JDSNMF algorithm does not consider the rich prior knowledge in multi-omics data, which can induce the algorithm to select biologically meaningful modules (Wei et al., 2022).

To this end, this paper proposed a prior knowledge-driven joint, semi-nonnegative matrix factorization algorithm (PD-JDSNMF) to integrate miRNA, mRNA, and lncRNA. The algorithm adds PPI interaction information based on the JDSNMF algorithm. The aim is to drive the algorithm to obtain more biologically meaningful co-expression modules. Since the algorithm based on matrix factorization is more sensitive to noise, we add Laplacian matrix constraints on the three kinds of data to enhance the anti-noise performance of the algorithm. We used the PD-JDSNMF algorithm to obtain multiple biologically significant co-expression modules. For the modules with strong correlation, we performed correlation analysis on the three types of data and mined multiple pathways significantly related to the disease. In addition, we constructed a heart failure diagnosis model using multiple classifiers for the top 13 features with high importance in this module, and its AUC in the test set reached the highest of 0.8714. We use an independent external validation set for diagnostic model validation, which achieves the highest AUC of 0.8329. It suggests that the selected features have diagnostic significance for heart failure.

## Meterial and methods

**FIGURE 1:**
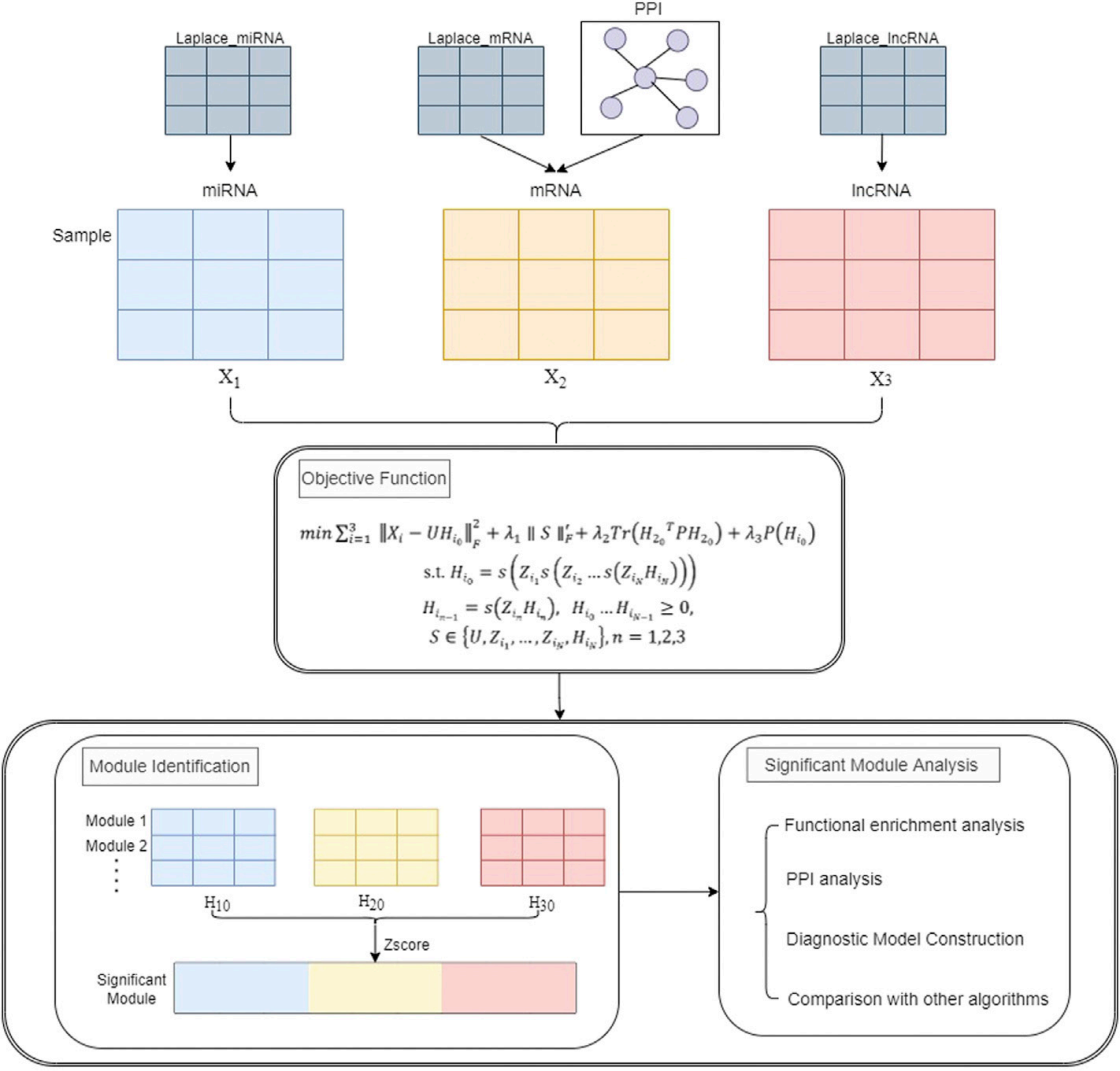
The overall flow chart of the experiment.

This section describes the framework of the PD-JDSNMF algorithm, which combines three types of RNA-seq data to identify co-expression modules. Figure 1 below presents the overall experimental framework of this paper. First, the objective function and its iterative update rule are given. The input of the whole framework consists of three parts, one is the expression profile of miRNA, mRNA, and lncRNA of the same set of samples (represented as matrix $X_{1}$ , $X_{2}$ , $X_{3}$ ), and the second part is the PPI prior knowledge, which is used to describe whether it has an interactive relationship between mRNA and mRNA. The third part is the Laplacian matrix of the three RNAs, which is used to improve the anti-noise performance of the algorithm.

Then, the three RNA expression matrices are decomposed into a common basis matrix W and three coefficient matrices $H_{1}$ , $H_{2}$ , and $H_{3}$ by the PD-JDSNMF algorithm. Membership in the co-expression module is confirmed based on the z-score of the resulting coefficient matrix after decomposition. Finally, further analysis is performed using elements from modules with strong correlations, including exploring co-expression modules associated with HF and analyzing significant pathways and key genes and constructing age-related regression models to identify significant potential correlations with patient age. The validity of the proposed algorithm is verified using genes and the construction of a diagnostic model of HF.

### Joint non-negative matrix factorization

Let $X_{i}\in R^{n\times p_{i}}$ represent the original matrix of different modes, $X_{i}$ is decomposed into a base matrix $U\in R^{n\times k}$ , and coefficient matrix $H_{i}\in R^{k\times m_{i}}$ , JNMF algorithm Decompose $X_{i}$ into a common base matrix U and multiple coefficient matrices $H_{i}\left(i=1,2,\ldots \right)$ , and define its objective function as follows:

$$\begin{array}{c} \Gamma \left(W,H_{i}\right)=\min_{W,H}\left(\overset{N}{\underset{i=1}{\sum }}{\left\|X_{i}-UH_{i}\right\|}_{F}^{2}\right)\;s.t.\;W>0,{\;H}_{i}>0,\;i=1,2,3,\ldots ,N. \end{array}$$ (1)

### Joint Deep Semi-Negative Matrix Factorization

JDSNMF adopts the principle of multilayer NMF and nonlinear activation functions to represent nonlinear manifolds. Also, it uses regularization to prevent overfitting. Its objective function is as follows:

$$\begin{array}{c} \begin{array}{c} \min\overset{I}{\underset{i=1}{\sum }}\;{∥X_{i}-UH_{i_{0}}∥}_{F}^{2}+\lambda ∥S{∥}_{F}^{\prime }\\ \;s.t.\;H_{i_{0}}=s\left(Z_{i_{1}}s\left(Z_{i_{2}}\ldots s\left(Z_{i_{N}}H_{i_{N}}\right)\right)\right)\\ H_{i_{n-1}}=s\left(Z_{i_{n}}H_{i_{n}}\right),H_{i_{0}}\ldots H_{i_{N-1}}\geq 0,\\ S\in \left\{U,Z_{i_{1}},\ldots ,Z_{i_{N}},H_{i_{N}}\right\},n=1,\ldots ,N \end{array} \end{array}$$ (2)

Among them, $U\in R^{n\times k_{0}}$ is called the sample latent matrix, $H_{i_{0}}\in R^{k_{0}\times p_{i}}$ is called the feature latent matrix of the first layer neural network, $H_{i_{n}}\in R^{k_{n}\times p_{i}}$ is the feature latent matrix of the sublayer n+1 . $Z_{n}\in R^{k_{n-1}\times k_{n}}$ is the junction latent matrix. In the JDSNMF algorithm, $k<\min\left\{n,p_{i}\right\}$ needs to be satisfied, and $k_{0}<k_{n}$ . Furthermore, they used the sigmoid activation function in the neural network to decompose H nonlinearly. The expression for this activation function is given below.

$$\begin{array}{c} s\left(x\right)=\frac{1}{1+e^{-x}}. \end{array}$$ (3)

### PD-JSNMF

This section proposed a prior knowledge-driven Joint Deep Semi-Non-Negative Matrix Factorization. Specifically, we added PPI interaction information to the feature latent matrix $H_{i_{0}}$ in the first layer of the algorithm, aiming to induce the algorithm to generate more biologically meaningful co-expression modules. Furthermore, we imposed a Laplacian constraint on $H_{i_{0}}$ to resist noise in the multi-omics data. The objective function of PD-JDSNMF is given below.

$$\begin{array}{c} \begin{array}{c} \min\overset{3}{\underset{i=1}{\sum }}\;{∥X_{i}-UH_{i_{0}}∥}_{F}^{2}+\lambda_{1}∥S{∥}_{F}^{\prime }+\lambda_{2}Tr\left(H_{2_{0}}^{T}PH_{2_{0}}\right)+\lambda_{3}P\left(H_{i_{0}}\right)\\ \;s.t.\;H_{i_{0}}=s\left(Z_{i_{1}}s\left(Z_{i_{2}}\ldots s\left(Z_{i_{N}}H_{i_{N}}\right)\right)\right)\\ H_{i_{n-1}}=s\left(Z_{i_{n}}H_{i_{n}}\right),\;H_{i_{0}}\ldots H_{i_{N-1}}\geq 0,\\ S\in \left\{U,Z_{i_{1}},\ldots ,Z_{i_{N}},H_{i_{N}}\right\},n=1,2,3 \end{array} \end{array}$$ (4)

Among them, $P\in R^{p_{2}\times p_{2}}$ is the PPI prior knowledge matrix with only two elements, 0 and 1. One means mRNA-mRNA interaction, and 0 means no interaction. In addition, a Laplacian matrix is introduced in the algorithm as a penalty term to improve the similarity of the related elements of the norm vector. This constraint forces the corresponding canonical correlation coefficient vectors to be more similar when the connectivity between the i−th node and the j−th node of the data is high (Kim et al., 2019). Its expression is as follows.

$$\begin{array}{c} P\left(H_{i}\right)=\underset{p,q}{\sum }L_{H_{i}\left(p,q\right)}\left(H_{ip}-H_{iq}\right)\left(i=1,2,3\right) \end{array}.$$ (5)

Here, $L_{h_{1}},L_{h_{2}},L_{h_{3}}$ represent the connectivity matrices of $X_{1}$ , $X_{2}$ and $X_{3}$ , respectively. Next, we further rewrite $P\left(H_{i}\right)\;\left(i=1,2,3\right)$ into the following form:

$$\begin{array}{c} P\left(H_{i}\right)=Tr\left(H_{i_{0}}^{T}B_{i}H_{i_{0}}\right), \end{array}$$ (6)

$B_{i}$ represents the Laplace matrix of $X_{i\;}\left(i=1,2,3\right)$ and $B_{i}=D_{i}-L_{i}\;\left(i=1,2,3\right)$ , $D_{i}$ respectively represents the degree matrix of the three kinds of data, and $L_{i}$ respectively represents the connectivity matrix of the three kinds of data.

### Protein-protein Interaction Network construction

The STRING (http://string-db.org) database was used to predict the PPI network in the module and analyze the interactions between proteins. Pairs of nodes in the PPI network were screened, and interactions with a combined score >0.4 were considered statistically significant. Molecular interaction networks were visualized using Cytoscape. Next, we identified core genes from the PPI network using the MCC method in cytoHubba (Cytoscape plugin).

### Functional enrichment analysis

To explore the biological functions of genes in modules in HF, we performed Disease ontology (DO) enrichment analysis and Kyoto Encyclopedia of Genes and Genomes (KEGG) enrichment analysis in selected modules. The DO enrichment analysis as well as the KEGG enrichment analysis were performed by the “clusterProfiler” package. The “ggplot” package is used to draw bubble plots. In DO analysis, q-values less than 0.05 were considered statistically significant. Pathways with *p*-values less than 0.05 were considered significant in the KEGG analysis.

## Results

### Data preprocessing

We downloaded RNA-seq data (GSE141910) containing 200 HF samples and 166 normal samples from the GEO database (https://www.ncbi.nlm.nih.gov/geo/). This paper used the human genome assembly GRCh38 file to convert the ensemble ID of GSE141910 data into gene name and genotype annotation and extracted 14914 mRNAs, 3,134 lncRNAs, and 30 miRNAs. Moreover, we downloaded the GSE116250 data from the GEO database as a validation dataset for ROC analysis. 14 normal samples and 50 HF samples were included in the GSE116250 data. Then randomly select 80% of the samples as the training set and 20% as the test set. Finally, 292 training samples and 74 testing samples are obtained.

**FIGURE 2:**
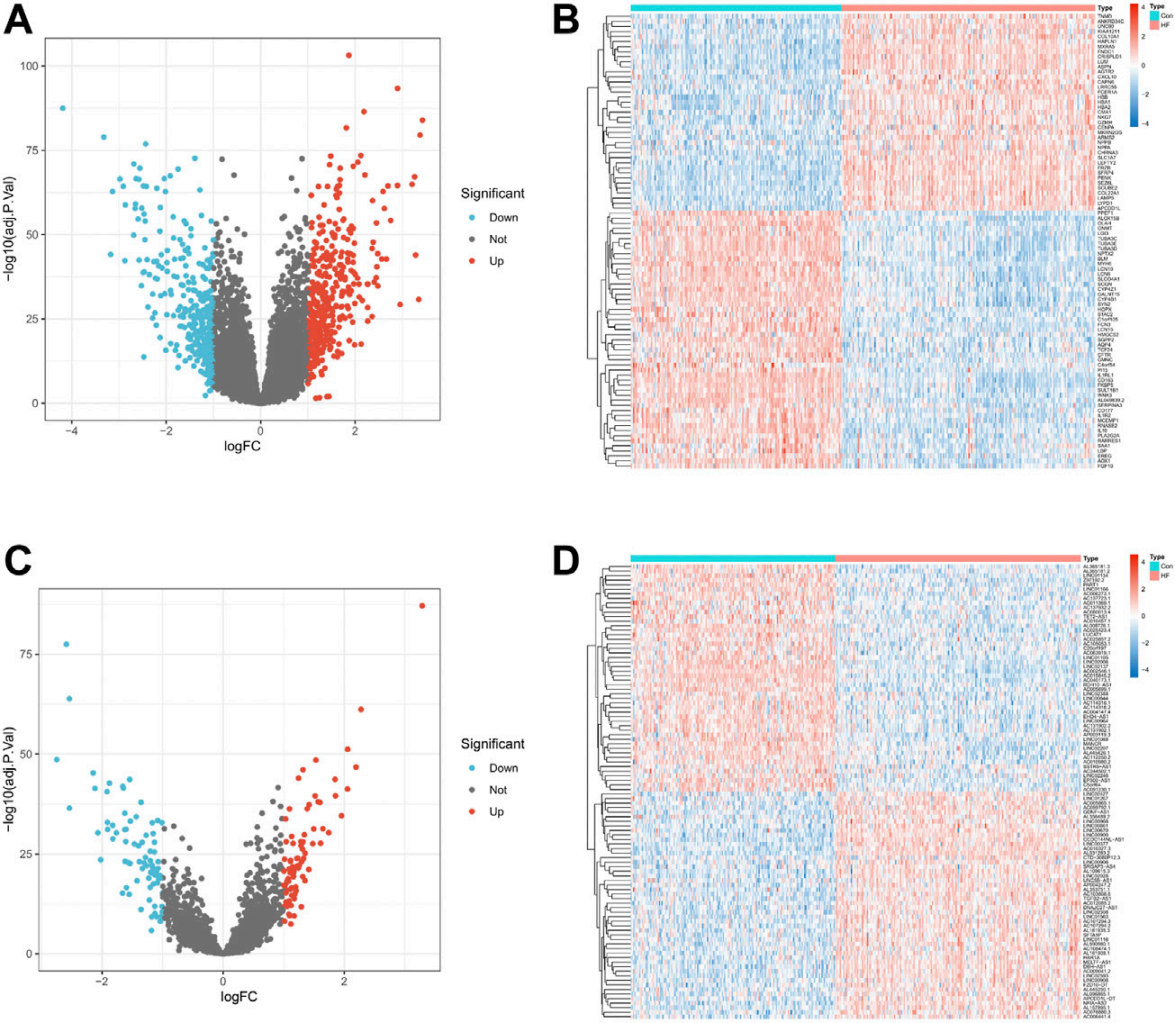
Differential expression levels of mRNA and lncRNA in HF. **(A,C)**. Volcano plot of 727 DEmRNAs and 162 DElncRNAs. Up-regulated genes are indicated in red, and down-regulated genes are indicated in blue. Genes with no significant change are marked as grey dots. **(B,D)**. Expression heat map of Top 100 DEmRNA and Top 100 DElncRNA. Red means genes are up-regulated, blue means genes are down-regulated.

We normalized the three RNA expression data using the R package “limma” and performed differential expression analysis on the expression data of mRNA and lncRNA. mRNAs or lncRNAs whose absolute value of logFC was less than 1 and *p*-value less than 0.05 were regarded as differentially expressed genes. The “pheatmap” package draws volcano plot and heatmaps. Finally, this study obtained 727 differentially expressed mRNAs (DEmRNAs) (Figures 2A,B) and 162 differentially expressed lncRNAs (DElncRNAs) (Figures 2C,D).

### Hyperparameter settings

In this paper, the sigmoid function is used as the activation function so that the decomposed feature latent matrix has non-negative nonlinearity. Furthermore, for the initialization of the basis and coefficient matrices, we use the singular value decomposition (SVD) algorithm, which has been shown to produce better local optima for matrix classification algorithms.

**FIGURE 3:**
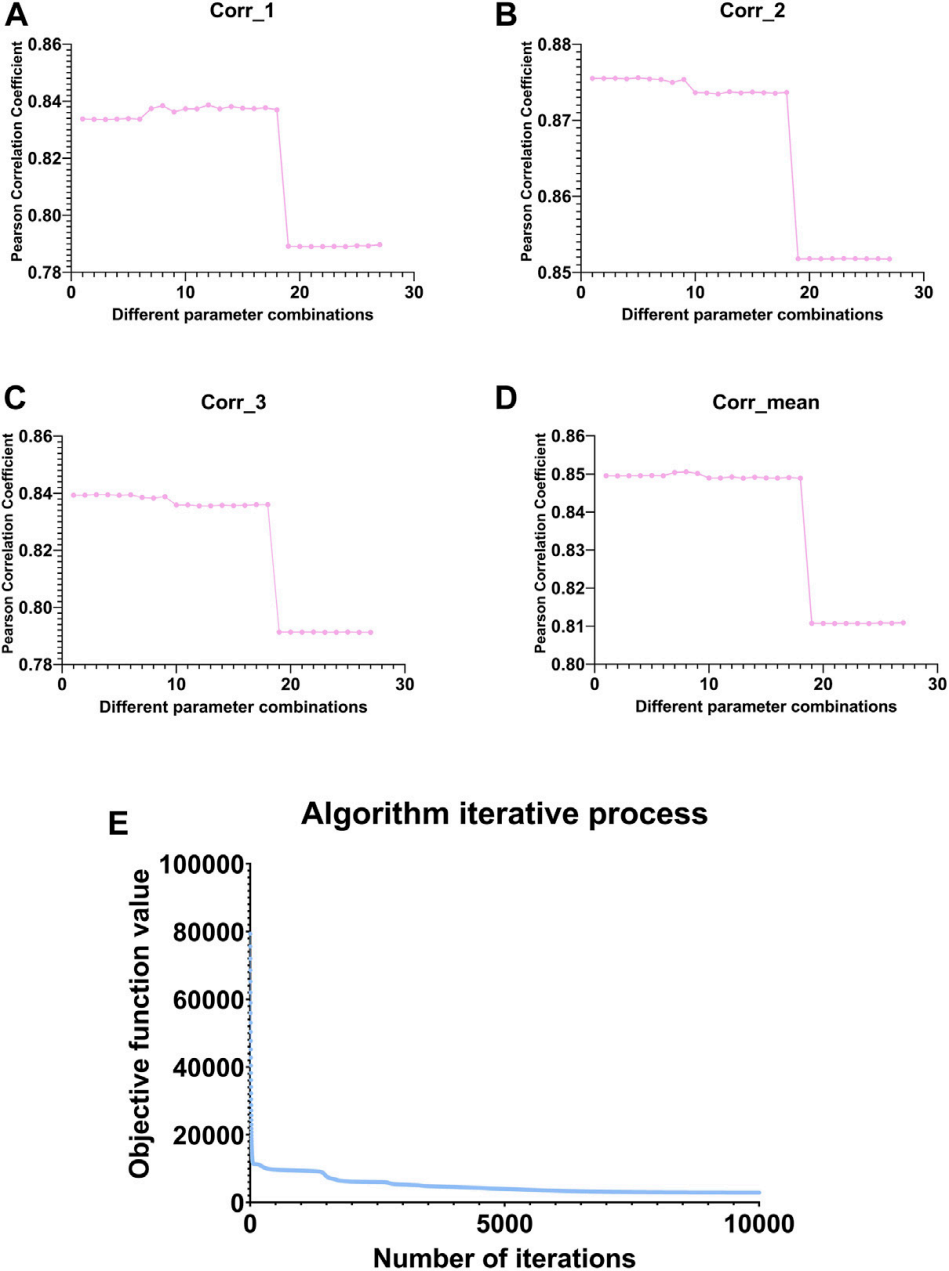
Line graph of the change in Pearson correlation coefficient between the original and reconstructed matrices under different parameter combinations. **(A–C)** represent the variation of the Pearson correlation coefficient between $X_{i}$ and $UH_{i_{0}}$ under different parameter combinations, respectively. **(D)** represents the mean of these three data under different parameter combinations. The circled point in each subplot represents the maximum value of the Pearson correlation coefficient for the group. **(E)** represents the changing trend of the algorithm’s objective function with the increase of the number of iterations in the training process under the optimal parameters.

Furthermore, this section will introduce the hyperparameter settings of PD-JDSNMF and classification models. PD-JDSNMF model has four key hyperparameters: number of layers, dimensionality reduction $k_{i}$ per layer, F-norm strength $\lambda_{1}$ , PPI prior constraint strength $\lambda_{2}$ , and Laplacian constraint strength $\lambda_{3}$ . In order to simplify the parameter selection process, we select these three hyperparameters from the parameter set para=[0.001 0.01 0.1] , a total of 27 parameter combinations. As shown in Figure 3, we use the Pearson correlation coefficient between the original matrix and the reconstructed matrix before and after matrix decomposition as the parameter evaluation index. Specifically, the larger the Pearson correlation coefficient, the better the reconstruction performance of our algorithm is considered to be.

We selected the parameters based on the mean value in subgraph D of Figure 3 and finally select the eighth group of parameters, whose corresponding parameters are $\lambda_{1}=0.001{,\lambda }_{2}=0.1,\lambda_{3}=$ 0.01. In addition, we invoked python’s scikit-learn library (Pedregosa et al., 2011) to evaluate the proposed algorithm’s feature selection ability and subsequent diagnostic model building. Specifically, we used four classification models: Random Forest (RF), Support Vector Machine (SVM), Logistic Regression (LR), and Deep Neural Network (DNN). When classifying ourselves, we use ten-fold cross-validation to select the critical parameters of the classifier. For RF, we set the number of decision trees between 1 and 200, and the attribute division methods are Gini and Entropy. For SVM, we set the penalty coefficient between 0 and 3, and the kernel function is selected from linear kernel function, polynomial kernel function, radial basis kernel function, and sigmoid function. For LR, we set the penalty term selected from $l_{1}$ and $l_{2}$ . The regularization coefficient is selected between 0 and 3. For DNN, we set the number of hidden layer neurons between 10 and 30. The activation function is selected from ReLU and Tanh, and the optimizer is selected from the stochastic gradient optimizer and chance stochastic gradient optimizer. The regularization parameters are chosen between 0.001 and 0.01. We set the same random seed for the above classifiers. Finally, we evaluate the classification results of several classifiers on the test set. In addition, we used DNN, LR, SVM, and RF four classifiers to classify three etiologies, including tonic cardiomyopathy (166 patients), hypertrophic cardiomyopathy (28 patients), and perinatal cardiomyopathy (6 patients). The results show that using the LR classifier for the top 9 features (MIR570, MIR590, MIR4506, MIR4786, IL1RL1, RNASE2, CD163, ST6GALNAC3, SIGLEC9) can achieve a classification accuracy of 0.9, which is higher than the classification results of other classifiers, showing the best classification performance. We put this part into the supplementary materials (Supplementary Figure S2).

### K value selection

In all matrix factorization models, dimensionality reduction k is a critical hyperparameter. If k is set too small, the data does not fit the model well. On the other hand, overfitting will occur if k is too large. From the experience of the JDSNMF algorithm, we set the number of network layers to four. Setting too many layers can cause the network to overfit the training data. Conversely, setting too few layers will result in insufficient network training. Since the smallest feature dimension in this experiment is 30, the $k_{0}$ of the first layer is set to 30. When setting $k_{1}$ and $k_{2}$ , we tried various cases ( $k_{0}$ , $k_{1}$ , and $k_{2}$ equal interval/unequal interval decay) Pearson correlation coefficient between the original data decomposed by the PD-JDSNMF algorithm the reconstruction matrix. As shown in Table 1, this indicator is one of the indicators that can best measure the reconstruction ability of the matrix factorization algorithm.

**TABLE 1:** Reconstruction performance of the algorithm under different k values in the neural network layer.

$k_{1}$	$k_{2}$	$k_{3}$	Corr_1	Corr_2	Corr_3	Corr_mean
$k_{1}$ = 29	$k_{2}$ = 28	$k_{3}$ = 27	0.8170	0.8897	0.8461	0.8509 ± 0.0299
$k_{1}$ = 29	$k_{2}$ = 27	$k_{3}$ = 25	0.8169	0.8892	0.8459	0.8507 ± 0.0297
$k_{1}$ = 28	$k_{2}$ = 25	$k_{3}$ = 21	0.8223	0.8847	0.8507	0.8507 ± 0.0258
$k_{1}$ = 28	$k_{2}$ = 26	$k_{3}$ = 24	0.8165	0.8869	0.8464	0.8499 ± 0.0288
$k_{1}$ = 27	$k_{2}$ = 23	$k_{3}$ = 18	0.8275	0.8813	0.8418	0.8502 ± 0.0227
$k_{1}$ = 27	$k_{2}$ = 24	$k_{3}$ = 21	0.8218	0.8843	0.8434	0.8498 ± 0.0259
$k_{1}$ = 26	$k_{2}$ = 21	$k_{3}$ = 17	0.8306	0.8791	0.8399	0.8499 ± 0.0210
$k_{1}$ = 26	$k_{2}$ = 21	$k_{3}$ = 16	0.8308	0.8783	0.8402	0.8498 ± 0.0205
$k_{1}$ = 25	$k_{2}$ = 19	$k_{3}$ = 12	0.8365	0.8711	0.8354	0.8476 ± 0.0166
$k_{1}$ = 25	$k_{2}$ = 20	$k_{3}$ = 15	0.8362	0.8758	0.8383	0.8501 ± 0.0182

It can be seen from the above table that there are three sets of k values that can make the average reconstruction performance of the algorithm for the three matrices reach 0.85 or more. The 10th group obtained the slightest standard deviation, so we selected this group of k values for subsequent analysis. This paper also shows the changing trend of the objective function with the iterative update of the algorithm under the optimal parameter combination (Figure 3E). It can be seen that the algorithm converges very quickly.

### Module identification

After performing the PD-JNMF algorithm on the HF RNA-seq dataset with the optimal parameter combination, we obtained 30 co-expression modules, each of which contained an average of 5.47 miRNAs, 118.33 mRNAs, and 28.73 lncRNAs. The mean correlations of the reconstructed mRNA expression data, lncRNA expression data, and miRNA expression data with the original data were 0.8362, 0.8758, and 0.8383, respectively.

### Significant module analysis

We counted the Pearson correlation coefficients of the original and reconstructed matrices in all modules, and the Table 2 gives the details of the modules with the Top 3 Pearson correlation coefficients.

**TABLE 2:** Top 3 module details.

Module ID	Number of miRNA members	Number of mRNA members	Number of lncRNA members	Correlation
1	5	135	30	0.6851
12	4	144	32	0.4688
30	5	172	32	0.4067

**FIGURE 4:**
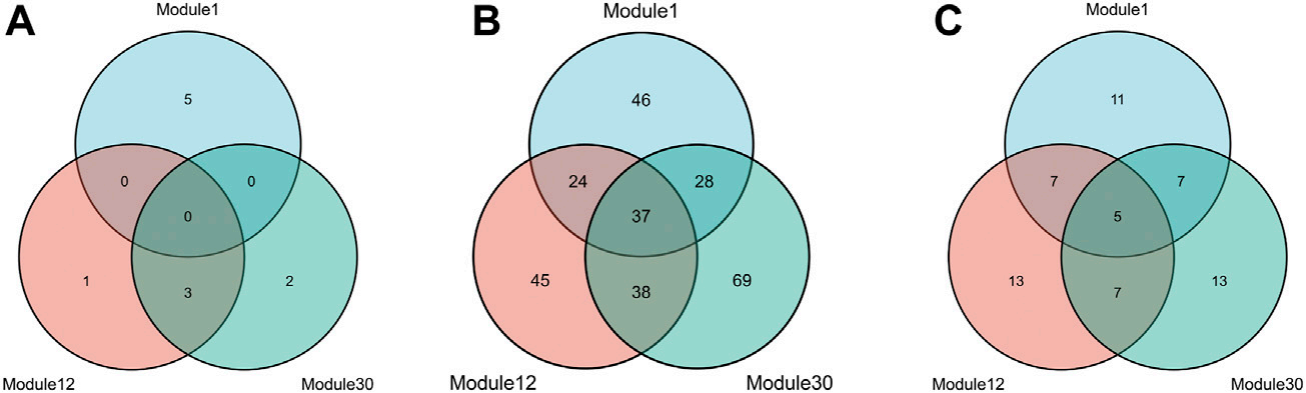
Three kinds of members Venn diagrams of the top 3 modules. The intersection of miRNAs **(A)**, mRNAs **(B)** and lncRNAs **(C)** in module 1, module 12 and module 30.

To meet the needs of subsequent analysis, we draw Venn diagrams for the three elements in these modules, as shown in Figure 4.

As shown from the Figure 4, miRNAs and mRNAs in module 12 have more intersections with the other two modules, suggesting that this module is more likely to be involved in complex mechanisms related to HF. Therefore, further analysis of module 12 will be carried out subsequently. In addition, this paper also draws a heat map of the expression levels of the three RNAs in module 12 on the training and test sets (Supplementary Figure S1).

### Biological analysis

**FIGURE 5:**
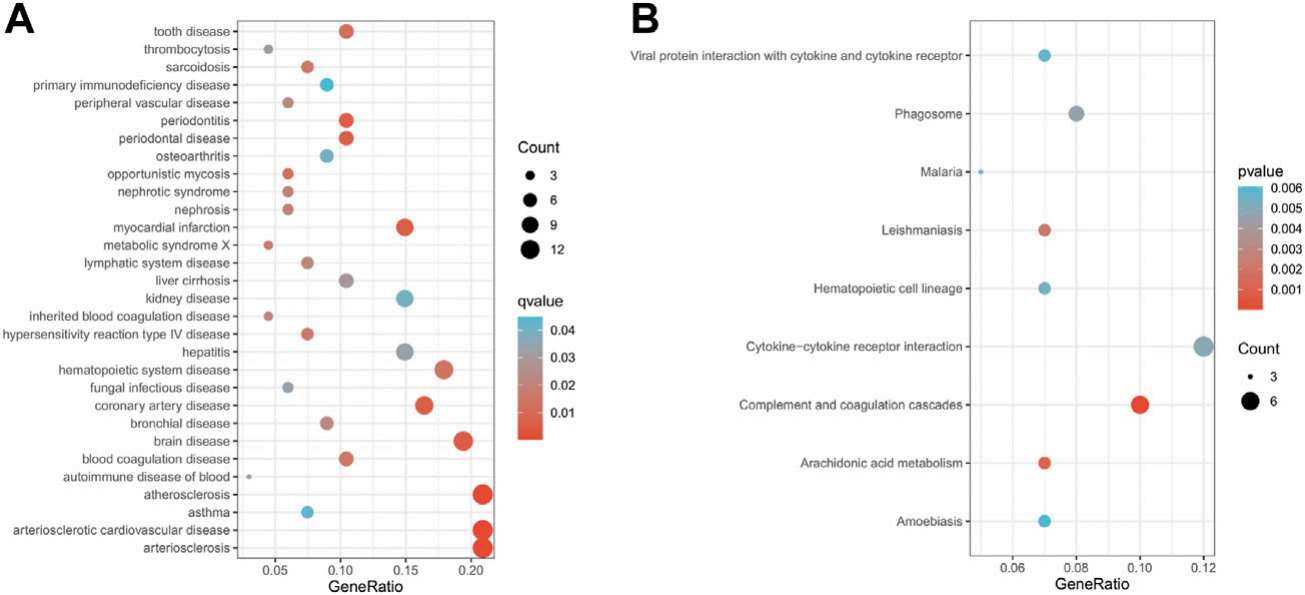
Enrichment analysis results of module 12 **(A)**. The results of DO enrichment analysis. **(B)** The results of KEGG enrichment analysis. The size of circles represented the number of genes enriched.

Subsequently, to explore the biological functions of genes in module 12, we perform DO enrichment analysis and KEGG enrichment analysis on module 12. The results of DO enrichment analysis show that the genes in module 12 are mainly associated with cardiovascular diseases, such as atherosclerosis, arteriosclerotic cardiovascular disease, arteriosclerosis, myocardial infarction, and coronary artery disease (Figure 5A). These diseases are risk factors for heart failure. KEGG enrichment analysis showed that the genes in module 12 were mainly involved in Complement and coagulation cascades, Cytokine-cytokine receptor interaction, Arachidonic acid metabolism, and Leishmaniasis, Phagosome and Hematopoietic cell lineage (Figure 5B). These pathways are closely related to HF. Studies have shown that Complement and coagulation cascades are involved in the post-MI response (Yin et al., 2022). Many studies have found that Cytokine-cytokine receptor interaction plays an essential role in the occurrence and development of acute myocardial infarction and HF (). Danqi Pill can prevent heart failure by regulating the pathway of Arachidonic acid metabolism (Wang et al., 2014). The above results suggest that the genes in module 12 may play an essential role in the pathological progression of HF.

**FIGURE 6:**
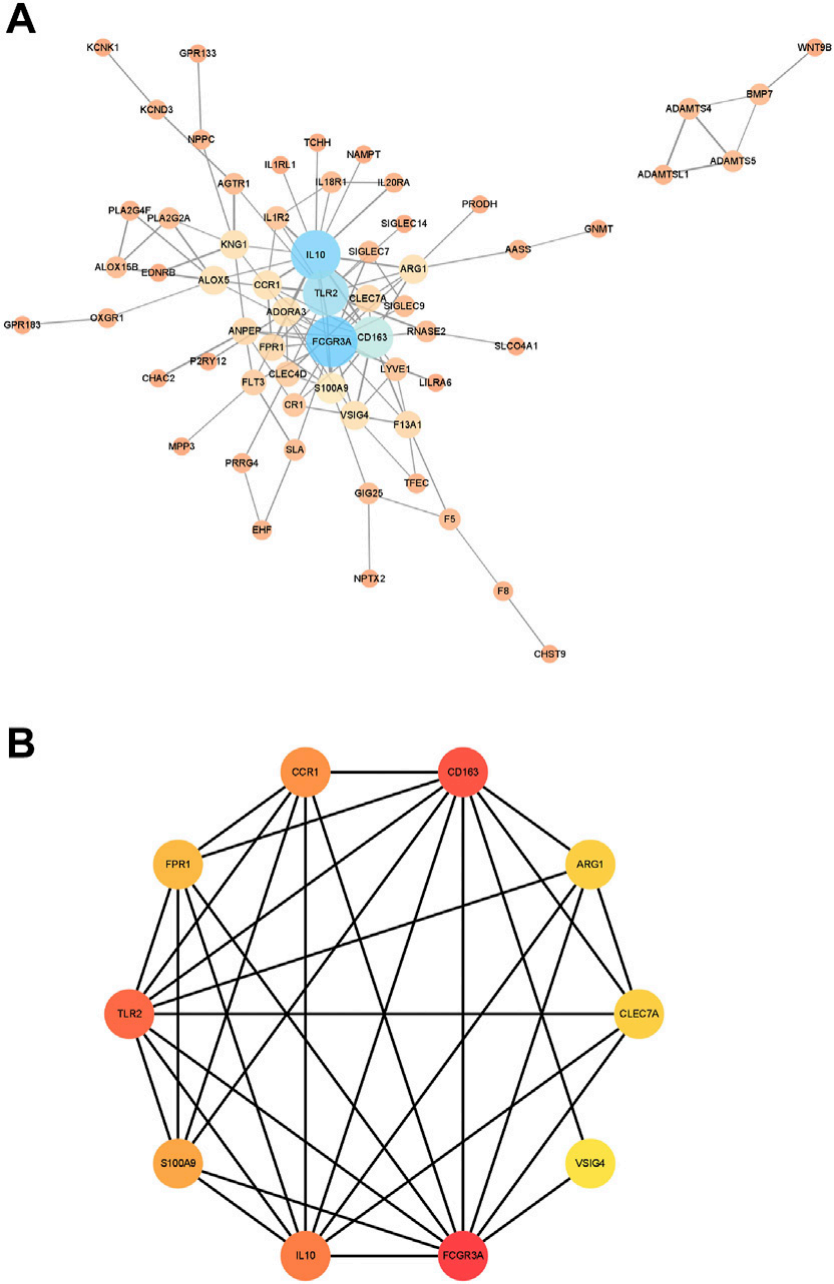
The results of PPI network. **(A)**. The PPI network were analyzed using NetworkAnalyzer plugin. **(B)**. The hub genes with the top 10 scores. The size and color of the nodes represent the importance of genes in the interaction network. The larger the node or the darker the color, the more important the corresponding gene is in the network. The connection between the nodes represents the interaction between the two genes, and the wider the connection line, the stronger the interaction between the two genes.

Next, we constructed a PPI network for the genes in module 12 and evaluated the network using the NetworkAnalyzer plugin in Cytoscape (Figure 6A). Next, we used the MCC method in cytoHubba to identify important genes in the PPI network, and the top 10 scoring genes were identified as core genes of module 12 (Figure 6B). They are VSIG4, IL10, FPR1, FCGR3A, TLR2, ARG1, CLEC7A, CCR1, S100A9, CD163.

## Discussion

### Comparison with other algorithms

**FIGURE 7:**
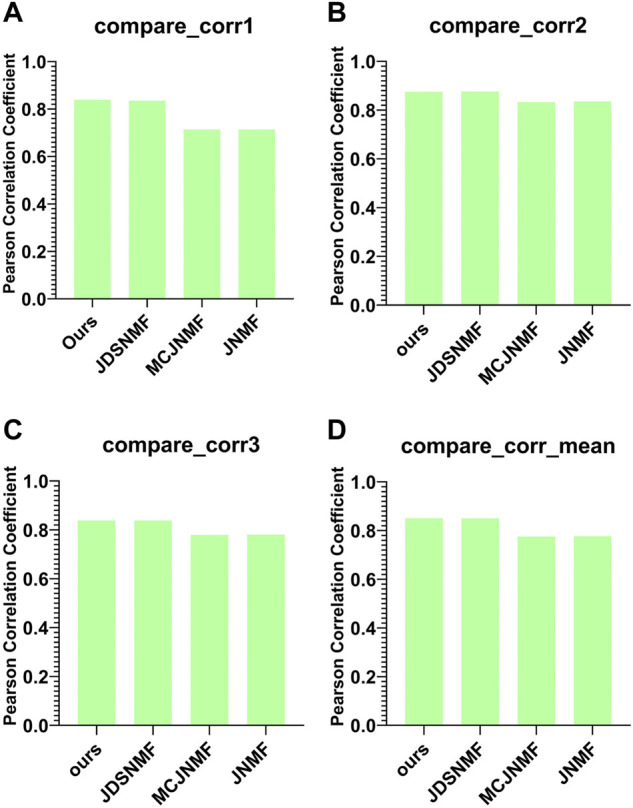
Comparison of reconstruction capabilities of the four algorithms. **(A–C)** are histograms of the Pearson correlation coefficients of the four algorithms between $X_{i}$ and $UH_{i_{0}}$ under the same experimental conditions **(D)**. The histogram of the mean comparison of the Pearson correlation coefficients in the first three graphs.

Figure 7 presents the reconstruction capability comparison between the proposed PD-JDSNMF algorithm and the other three NMF-based algorithms.

As can be seen from Figure 7, the two NMF algorithms based on nonlinear decomposition strategies achieve more robust matrix reconstruction performance. The average Pearson correlation coefficient of the proposed PD-JDSNMF algorithm is more significant than that of the other three algorithms, which verifies the effectiveness of the proposed algorithm to a certain extent.

### Construction of the diagnostic model

**FIGURE 8:**
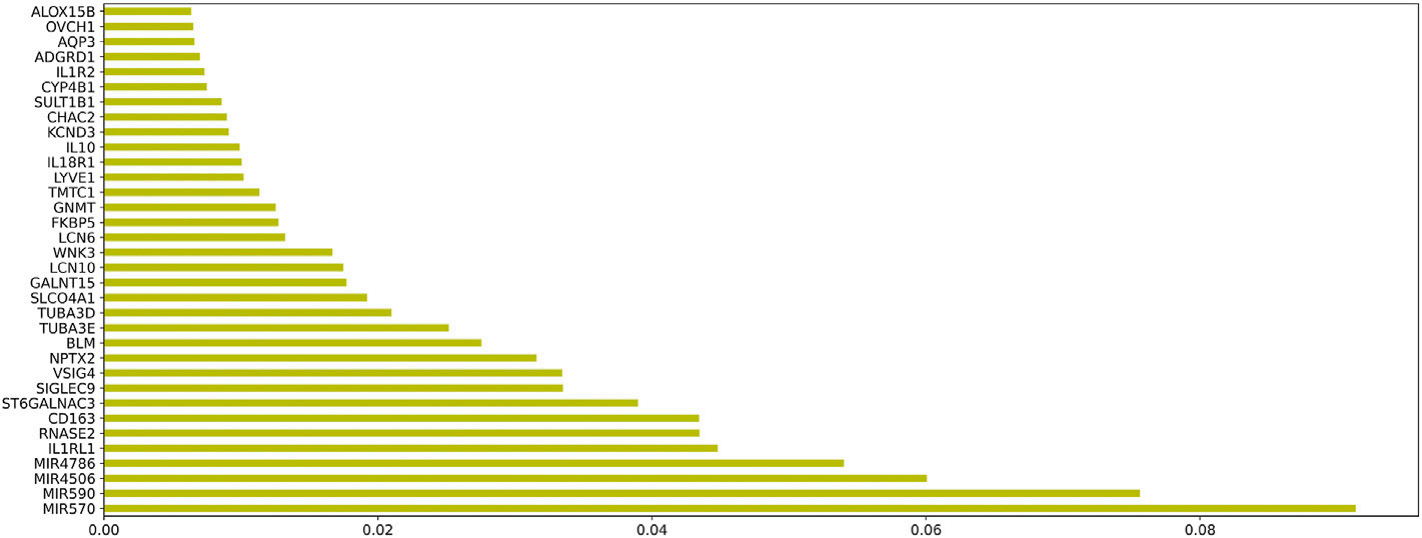
The histogram of the Top 50 genes and their corresponding weights in module 12.

In order to evaluate the genes in module 12 as having diagnostic significance for HF, we first used the RF algorithm to rank the feature importance in this module. Figure 8 shows the top 50 elements with solid importance in this module.

**FIGURE 9:**
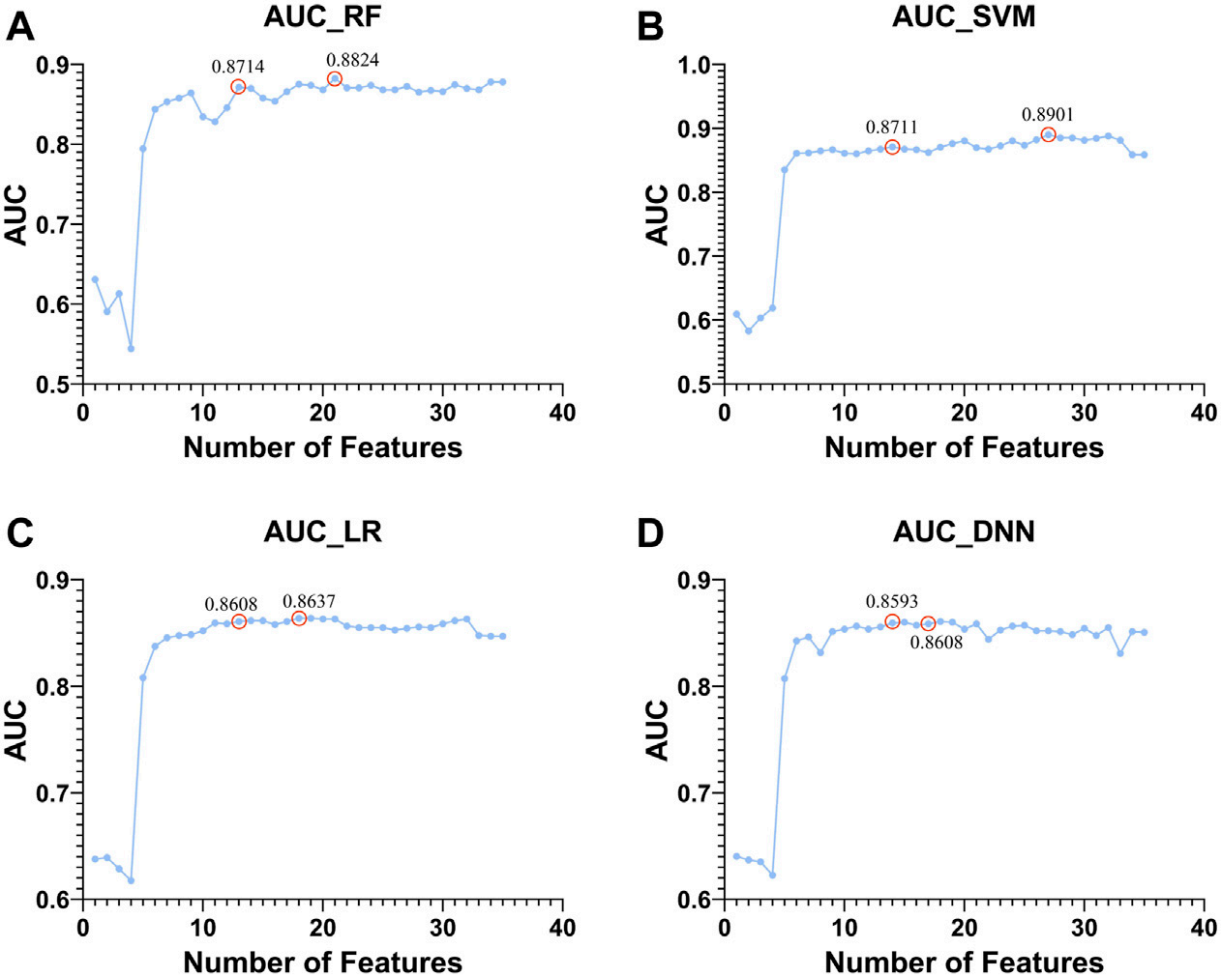
Top 1 to Top 50 features to classify whether subjects are sick or not and shows the trend of increasing AUC with the features used. The classifiers used by **(A–D)**. RF, SVM, LR, and DNN, respectively.

Next, based on four classifiers (RF, SVM, LR, and DNN), we used Top 1 to Top 50 genes in module 12 to classify whether there was HF or not and compared the changes in AUC as shown in Figure 9.

**FIGURE 10:**
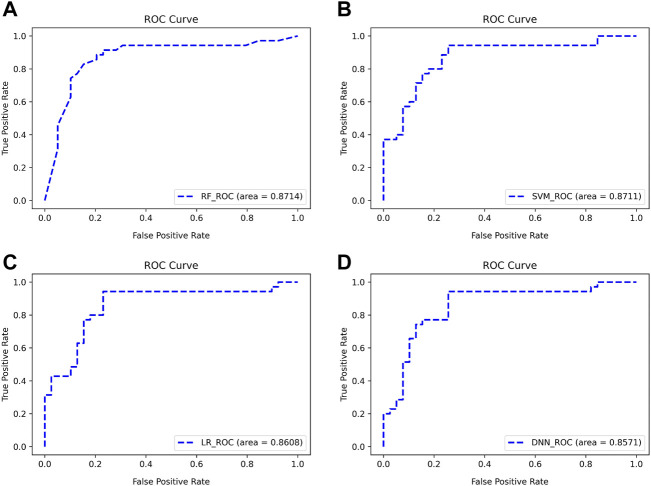
ROC curves for classifying subjects using Top features **(A–D)**. The classifiers used are RF, SVM, LR, and DNN, respectively.

There are two points marked in each subplot in Figure 9. The first point of each subgraph represents the case where the classifier is used to select as few features as possible to guarantee a high AUC. The second point represents where the AUC is maximized using this classifier. As can be seen from the figure, the maximum AUC is achieved with the RF classifier, which is 0.8824. In addition, when using this classifier to select the Top 13 features, the AUC can reach 0.8714. Therefore, RF has a better classification effect on this dataset than the other three classifiers. For the first point of the four classifiers, we plotted its ROC curve in detail, as shown in Figure 10.

**FIGURE 11:**
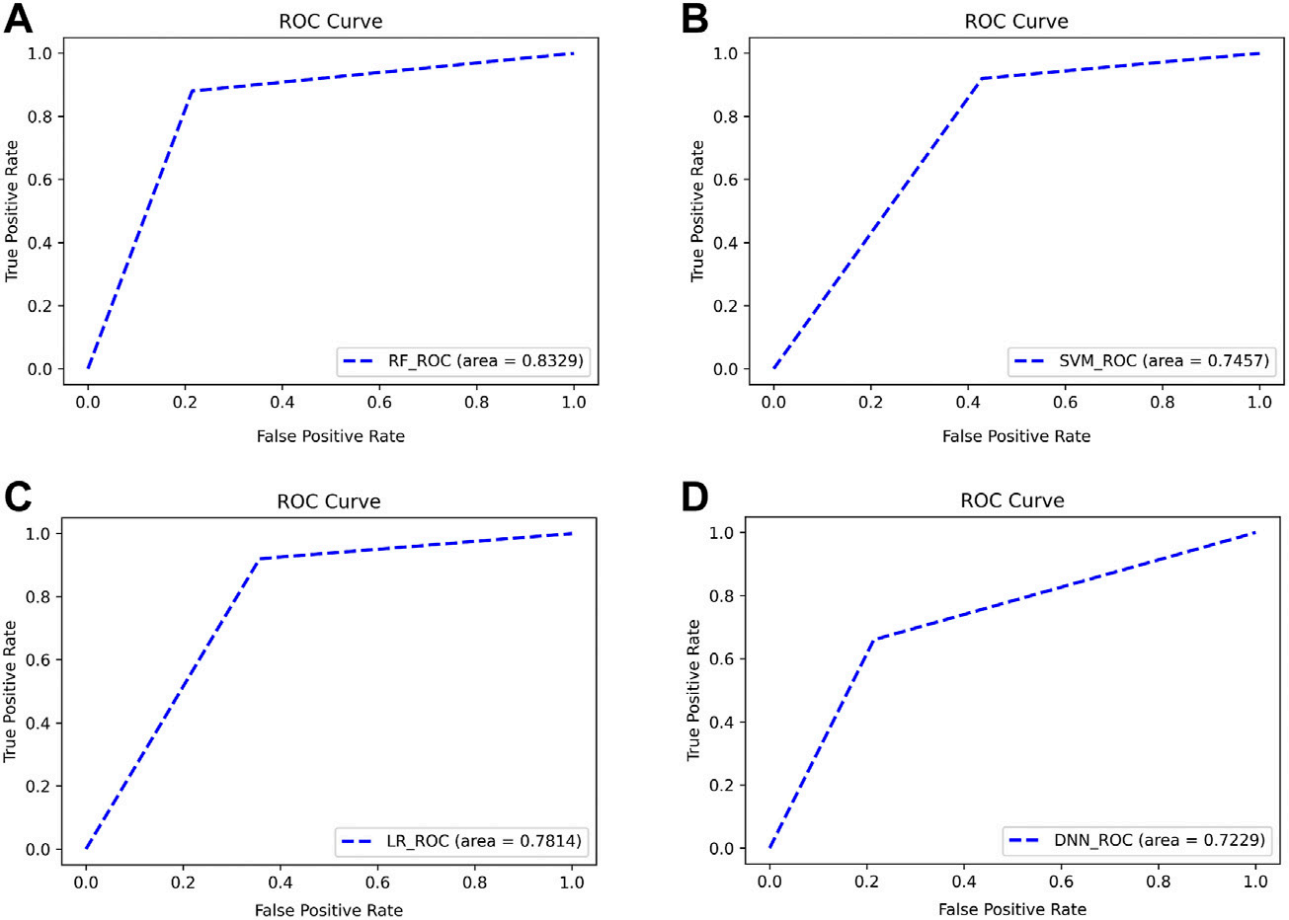
ROC curves for classifying subjects on external validation set using Top 13 features. The classifiers used by **(A–D)** are RF, SVM, LR, and DNN, respectively.

As can be seen from Figure 10, the four classifiers all have high classification accuracy at their respective first points. To further validate that the Top 13 genes have diagnostic significance for HF, we performed validation using the Top 13 genes in an external validation set. Figure 11 shows the ROC curve for this external validation set based on the four classifiers using Top 13 features.

As can be seen from Figure 11, using the Top 13 genes also obtained better classification accuracy on the external validation set. Among them, the AUC using the RF classifier reached 0.8329. This confirms that the Top 13 genes have a robust diagnostic ability for HF.

### Biological significance of genes involved in diagnostic models

This study identified 13 genes (MIR570, MIR590, MIR4506, MIR4786, IL1RL1, RNASE2, CD163, ST6GALNAC3, SIGLEC9, VSIG4, NPTX2, BLM, TUBA3E) that were closely related to HF. Myocardial infarction leads to the death of cardiomyocytes, leading to cardiac fibrosis, cardiac remodeling, and heart failure. In a human fibroblast model, researchers found that MIR590 inhibits cardiac fibrosis after myocardial infarction (Yuan et al., 2020). Chronic inflammation and fibrosis in the heart muscle eventually lead to heart failure. Studies have confirmed that MIR590 is closely related to myocarditis (Oh et al., 2022). CircRNA-0068481 can promote the pathological progression of right ventricular hypertrophy (VH) by regulating the expression of MIR570 (Guo and Liu, 2021). MiRNA-induced regulation can be propagated through transcription factors (TFs) (Naeem et al., 2011). STAT1 is one of the transcription factors of MIR4506. STAT1 can lead to the loss of cardiomyocytes by increasing apoptosis and reducing cardioprotective autophagy (Knight et al., 2012). CEBPB is one of the transcription factors of MIR4786. CEBPB inhibits cardiomyocyte growth and proliferation in the mammalian heart, and the reduction of CEBPB is a core signal of physiological hypertrophy and proliferation (Boström et al., 2010). Broch et al. (2012) found that L1RL1 can reflect the activity of the interplay of inflammation and hemodynamic stress in heart failure and may be a potential therapeutic target for heart failure. Idiopathic pulmonary arterial hypertension (IPAH) can lead to heart failure. In the experiment between IPAH and the control group, the researchers found that RNASE2 is involved in the pathogenesis of IPAH (Zeng et al., 2021). It has been reported that CD163 concentrations are elevated in heart failure patients compared with healthy controls (Ptaszynska-Kopczynska et al., 2016). A previous study identified VSIG4 as a right ventricle-specific myocardial biomarker (di Salvo et al., 2015). SIGLEC9 negatively regulates inflammatory responses. Inflammation is an essential factor in the development and progression of HF (Chaikijurajai and Tang, 2020). NPTX2 encodes a synaptic protein associated with C-reactive protein. Several studies have confirmed that C-reactive protein can effectively predict the occurrence of HF (Maio et al., 2021). Deletion or mutation of BLM may result in telomere defects and accelerated telomere shortening (Callén and Surrallés, 2004). Studies have shown that cardiomyocyte-specific telomere shortening is a striking feature of HF (Sharifi‐Sanjani et al., 2017). TUBA3E encodes α-tubulin and is closely associated with cytoskeletal remodeling. Microtubule accumulation was found in HF patients, thereby increasing the load on myocytes and promoting cardiac dysfunction (Hein et al., 20001072). The above results suggest that these 13 genes may play essential roles in the occurrence and progression of HF.

### Comparison with other algorithms

**FIGURE 12:**
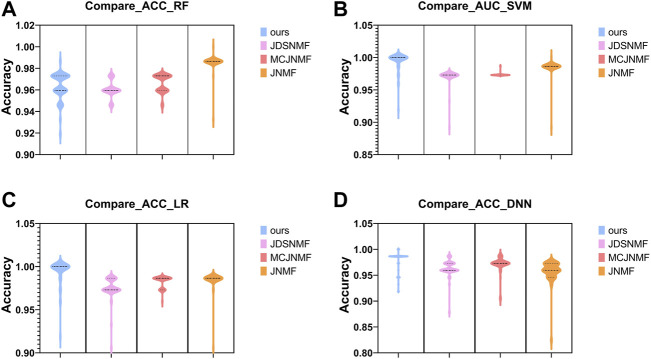
Violin plots for AUC using four classifiers to classify the features of the four algorithms in their respective significant modules. **(A–D)**. The comparison results obtained using RF, SVM, LR, and DNN.

In order to verify the ability of the proposed algorithm to reconstruct the original matrix, we use the features selected by the proposed PD-JDSNMF and the three algorithms of JDSNMF, MCJNMF, and JNMF, and use four classifiers to compare the classification accuracy. To fairly compare the feature selection ability of several algorithms, we first select the module with the most robust reconstruction performance from all co-expression modules of several algorithms. Then, 50 features were randomly selected from the saliency module and repeated ten times to calculate the AUC for classification using the four classifiers, respectively, and the following violin plot was drawn as shown in Figure 12.

As can be seen from Figure 12 the proposed algorithm achieves higher AUC among the four classifiers, which again confirms that incorporating prior knowledge into the nonlinear matrix factorization algorithm can obtain more representative features.

## Conclusion

This paper proposed a PD-JDSNMF algorithm to integrate prior information in genetic data, extract nonlinear features in genetic data, capture the underlying expression patterns of multiple data, mine heart failure-related biological markers, and build diagnostic models. Specifically, we identified module 12 as a key module and the genes in the module as inputs for subsequent analyses through functional enrichment analysis. Using multiple classifiers, we constructed a heart failure diagnostic model and validated the diagnostic model on an external dataset, which achieved an AUC of 0.8329. Compared with several other NMF-based algorithms, the proposed algorithm has a stronger matrix reconstruction ability. Furthermore, the PD-JDSNMF algorithm is confirmed to have a more stronger feature selection ability by using elements for classification.

## Data Availability

The datasets presented in this study can be found in online repositories. The names of the repository/repositories and accession number(s) can be found in the article/Supplementary Material.
