# Erratum: Integration of RNA molecules data with prior-knowledge driven Joint Deep Semi-Negative Matrix Factorization for heart failure study

**DOI:** 10.3389/fgene.2022.1095803

**Published:** 2022-11-22

**Authors:** Frontiers Production Office

**Affiliations:** Frontiers Media SA, Lausanne, Switzerland

**Keywords:** heart failure, biomarker, joint nonnegative matrix factorization, diagnostic model, machine learning

Due to a production error, there was an error in the **Affiliations**. Instead of “Department of Cardiology, Shanghai Jiao Tong University School of Medicine, Shanghai, China”, it should be “Department of Cardiology, Shanghai Sixth People’s Hospital Affiliated to Shanghai Jiao Tong University School of Medicine, Shanghai, China”.

The publisher apologizes for this mistake. The original version of this article has been updated.

