# Supplementary material for: Integration of RNA molecules data with prior-knowledge driven Joint Deep Semi-Negative Matrix Factorization for heart failure study
Source: Front Genet. 2022 Oct 10;13:967363. doi: 10.3389/fgene.2022.967363 (PMC9589260; doi:10.3389/fgene.2022.967363)
Supplement: Supplementary file 1 [file DataSheet1.docx]

# Supplementary material


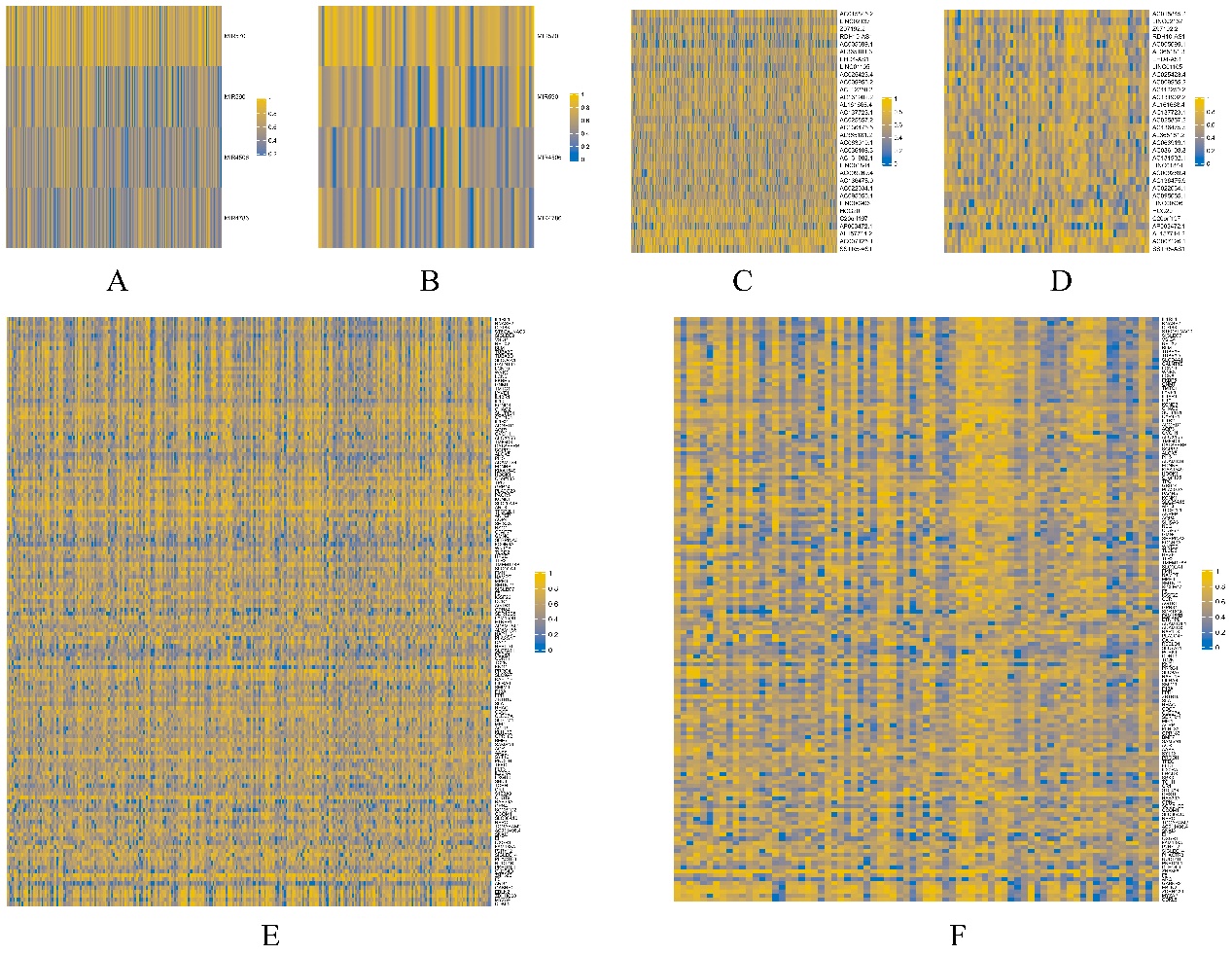


Fig.S1 Heatmap of expression of three RNAs in training and test samples in module 12. A, C, and E express miRNA, lncRNA, and mRNA on the training set. B, D, and F express miRNA, lncRNA, and mRNA on the test set.

Thanks to the multi-layer nonlinear transformation of the coefficient matrix by PD-JDSNMF, the PD-JDSNMF algorithm can identify the different patterns between the sick group and the normal group and the unique patterns between different causes. In addition to building a diagnostic model, we also use the features selected by the algorithm to classify HF patients with different etiologies. Specifically, the causes of 200 HF patients included in GSE141910 included dilated cardiomyopathy (166), hypertrophic cardiomyopathy (28), and perinatal cardiomyopathy (6). We use the features selected by PD-JDSNMF and DNN, LR, SVM, and RF classifiers to classify the above three causes, as shown in Figure S1.


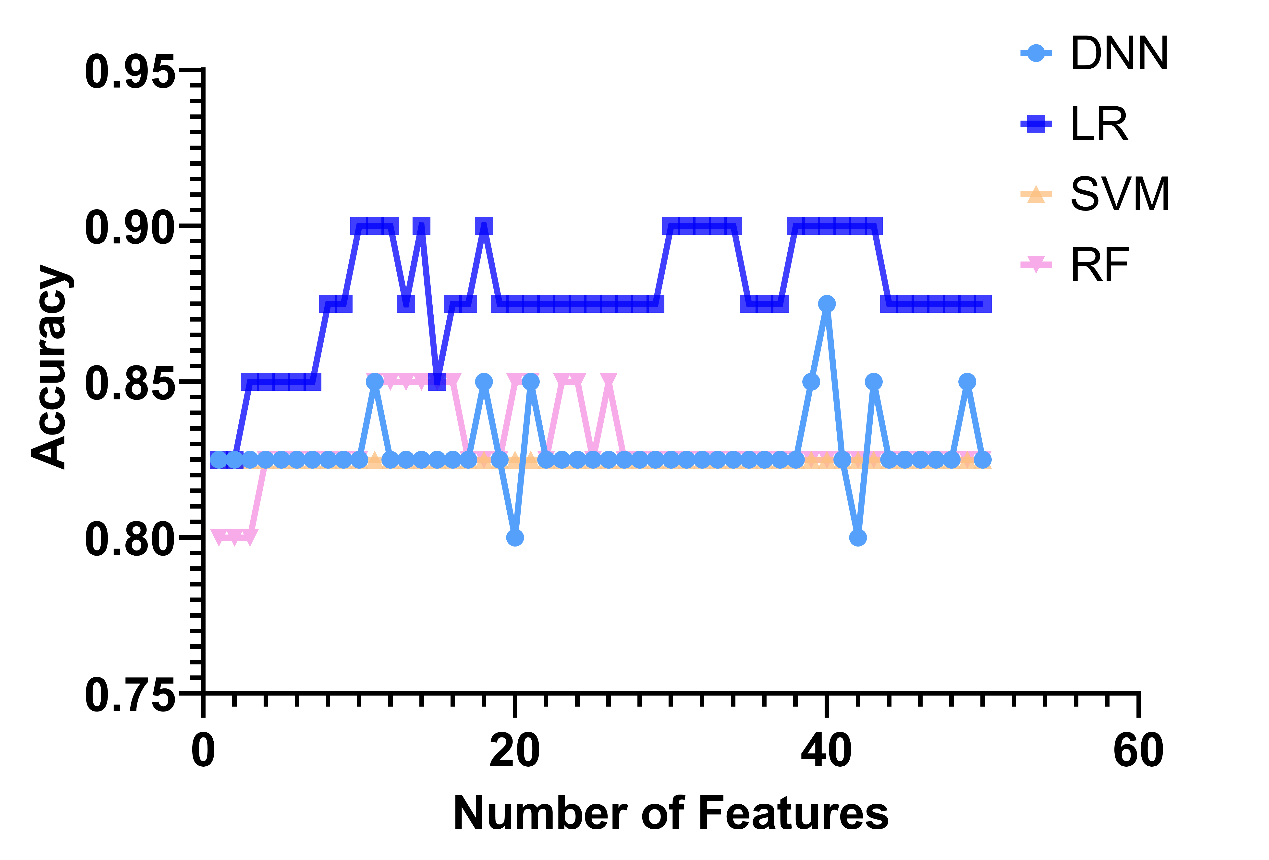


Fig. S2 Accuracy of HF etiology classification using Top 50 features selected by PD-JDSNMF algorithm.

As can be seen from Fig. S1, the LR classifier can achieve a classification accuracy of 0.9 for the Top 9 features (MIR570, MIR590, MIR4506, MIR4786, IL1RL1, RNASE2, CD163, ST6GALNAC3, SIGLEC9), which is higher than the classification results of other classifiers, showing the best classification performance.
